# Potential Role of Lipase Activity on the Internal Exposure Assessment of Glycidol Released from Its Fatty Acid Esters

**DOI:** 10.3390/toxics11020175

**Published:** 2023-02-14

**Authors:** Yuko Shimamura, Ryo Inagaki, Minami Oike, Yuri Wada, Hiroshi Honda, Shuichi Masuda

**Affiliations:** 1 School of Food and Nutritional Sciences, University of Shizuoka, 52-1 Yada, Suruga-ku, Shizuoka 422-8526, Japan; 2 R&D Safety Science Research, Kao Corporation, 2606 Akabane, Ichikai-Machi, Haga-Gun, Tochigi 321-3497, Japan

**Keywords:** glycidyl fatty acid esters, glycidol, lipase, glycidol–hemoglobin adduct (diHOPrVal), high-fat diet

## Abstract

Glycidyl fatty acid esters (GEs) can be found in food, and they can be converted into genotoxic animal carcinogen glycidol in vivo by the action of lipase. This study examined whether human ingestion of charbroiled pork containing high levels of GEs (300 µg/day) increased glycidol–hemoglobin adduct (diHOPrVal), a marker of internal exposure to glycidol using LC-MS/MS. Contrary to expectation, the diHOPrVal value before ingesting charbroiled pork was 3.11 ± 1.10 pmol/g globin, which slightly decreased to 2.48 ± 0.47 pmol/g globin after 5 days of consumption. The decrease in lipase activity caused by the continuous consumption of lipid-rich foods such as meat in humans might decrease internal exposure to glycidol released from its esters. Thus, lipase activity was measured in C57/BL6J mice fed a high-fat diet (HFD) for 8 weeks, and diHOPrVal formation was measured after the administration of glycidyl oleate. Lipase activity was significantly lower in the HFD group than in the normal diet group. The amount of diHOPrVal was reduced in the HFD group. Therefore, the lipase activity was reduced by HFD, thereby decreasing the degradation of glycidol from glycidyl oleate. These results indicate that changes in lipase activity depending on the amount of lipids in the diet may affect the assessment of GEs exposure, and monitoring the lipase activity would provide a comprehensive understanding of exposure assessment.

## 1. Introduction

**Figure 1:**
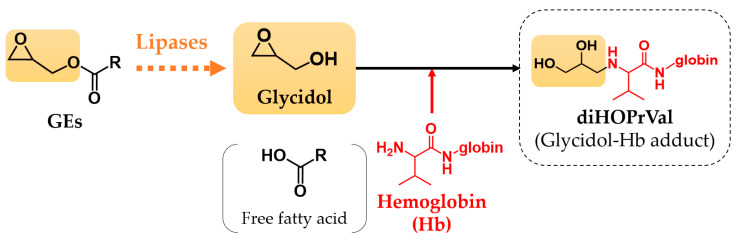
Formation of glycidol from glycidyl fatty acid esters (GEs) and glycidol–hemoglobin adduct (diHOPrVal).

Glycidyl fatty acid esters (GEs) are process contaminants formed mainly in the deodorization step under the high temperature conditions of the refining process of edible oil [1]. GEs, which have fatty acid in their structure, are degraded by lipase, a lipolytic enzyme, consequently producing glycidol (2,3-epoxy-1-propanol) when ingested by the body through the diet (Figure 1) [2]. Glycidol is known as a genotoxic epoxide compound and has been reported to induce carcinogenicity [3,4,5], chromosomal aberration induction, neurotoxicity, and mutagenicity [6,7,8,9]. In view of the genotoxic and carcinogenic potential of glycidol, it is important to assess the exposure of GEs to humans from the food and environment for public health perspective. *N*-(2,3-dihydroxypropyl)valine (diHOPrVal) is a hemoglobin (Hb) adduct of glycidol, which has been reported as an important external exposure marker for GEs [10,11,12,13]. The Hb adduct of glycidol is formed by the binding of glycidol to the *N*-terminal valine of the Hb structure (Figure 1) [14]. Honda et al. reported no significant increase in diHOPrVal levels among subjects who have ingested diacylglycerol (DAG) oil containing high levels of GEs [15,16]. This finding indicates that humans are exposed to GEs from sources other than DAG oil. Besides being food contaminants found at highest levels in refined vegetable oils and fats, GEs are found in cooked meat and fish [17], as well as prepared foods such as instant noodles, fried chicken, and fried confectionery as new sources [18]. In particular, high concentrations of GEs were detected in meat cooked on charcoal that can be heat treated at high temperatures. Landin et al. found that animals fed fried animal feed for 30 or 72 days showed an increase in diHOPrVal level by about 50% compared with controls [19]. Therefore, heating of food affected the formation of diHOPrVal. However, few human studies have measured adduct levels after ingesting foods containing GEs treated at high temperatures.

The release of glycidol from GEs is influenced by the action of lipases. Lipases are enzymes that hydrolyze fats, such as triacylglycerols and phospholipids, and separate fatty acids. Human lipases include preduodenal (tongue and stomach) and extraduodenal (pancreatic, liver, lipoprotein, and endothelial) lipases. Increased oxidative stress due to high-fat diet intake induces impaired lipid metabolism, leading to a decrease in lipase activity [20]. However, in the risk assessment of GEs, the extent to which glycidol is produced when GEs are hydrolyzed by lipases in vivo remains unclear. Lipase activity is altered by dietary components, and its decrease has been reported in the serum of rats fed a high-fat diet (HFD) [21]. Decreased lipase activity caused by increased serum lipid concentrations may reduce the efficiency of the conversion of GEs to glycidol. However, little is known about the relationship between lipase activity and GEs conversion efficiency to glycidol in vivo.

In this study, the amount of diHOPrVal was measured in subjects fed cooked pork containing GEs, and the relationship with GEs exposure was examined on the basis of diet and diHOPrVal levels. In addition, GEs were administered to mouse models of HFD-induced obesity, and the relationship between lipase activity and diHOPrVal formation was examined.

## 2. Materials and Methods

### 2.1. Chemicals

Glycidol (purity 96.0%) and fluorescein isothiocyanate (FITC; purity ≥ 90.0%) were obtained from Sigma-Aldrich (St. Louis, MO, USA). Glycidyl oleate was purchased from FUJIFILM Wako Pure Chemical Corporation (Osaka, Japan). L-*d*_8_-valine (purity 98.0%), which was used for the synthesis of the glycidol internal standard 3-(fluorescein-5-yl)-1-(2,3-dihydroxypropyl)-5-*d*_7_-isopropyl-2-thioxo-4-imidazolidinone (DHP-Val-*d*_7_-FTH) [12], was obtained from Cambridge Isotope Laboratories, Inc. (Tewksbury, MA, USA). Lipase Kit S was obtained from DS Pharma Biomedical Co., Ltd. (Osaka, Japan). All other chemicals and solvents used were of analytical grade.

### 2.2. Human Blood Sample Collection

The study was in accordance with the regulations of the University of Shizuoka ethical committee, including the obtainment of informed consent forms that explained the objectives of the study in advance (Permit Number 30-15). Twelve healthy men aged 20–40 years were selected as subjects. There are differences between men and women in blood test indices such as red blood cell count, hemoglobin level, hematocrit level, serum iron level, and ferritin level. Furthermore, menstrual bleeding causes differences in these indices among women. In addition, menstruation affects chemical metabolism. For these reasons, only men were included in this study. No dietary restrictions were imposed other than the test meal. As for smokers (3 persons), no difference in the average number of cigarettes smoked was observed before and after consumption of the test meal. As a test meal, pork belly (Aono Fresh Meats, Johoku, Japan) was heated over charcoal fire at over 400 °C for 3 min. The amount of GEs in the cooked pork was measured using the LC-MS method of a previous study [17]. The subjects took approximately 100 g/day of heated pork belly (weighing 250 g before heating) as a test meal for lunch and dinner for 5 days. The amount of GEs ingested from the test meal was approximately 300 µg/day. Blood samples were collected from the subjects using heparinized vacutainer tubes before and after the test. The donors were fasted for 12 h before blood was drawn.

### 2.3. Animals

**Figure 2:**
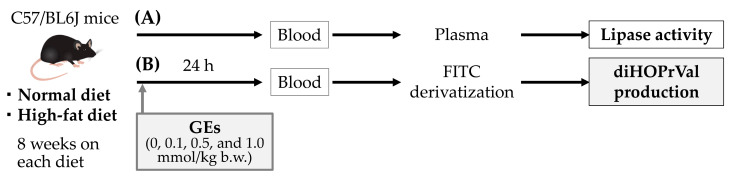
Procedures for animal experiments. (**A**) Lipase activity in plasma was measured in five normal diet and five high-fat diet groups. (**B**) Five normal diet and five high-fat diet groups were treated with 0, 0.1, 0.5, and 1.0 mg/kg b.w. of glycidyl fatty acid esters (GEs). Twenty-four hours after GEs administration, blood was drawn. After solid-phase extraction of blood samples, blood was derivatized using fluorescein isothiocyanate (FITC), and then diHOPrVal levels were measured by LC–MS/MS.

C57BL/6J mice (5 weeks old, ♂, Japan SLC) were purchased from Japan SLC Corporation (Hamamatsu, Japan) and reared in an animal center at 23 °C ± 1 °C with 55% ± 5% humidity under a 12 h light/dark cycle. After acclimation for 1 week, mice were divided into two groups: normal diet (*n* = 25) and HFD (*n* = 25) groups. The HFD group was fed a HFD (32% crude fat content; HFD32; Crea Japan, Tokyo, Japan) and weighed every other week for 8 weeks. By contrast, the normal diet group was fed CE-2 (4.8% crude fat content; Crea Japan). Water drinking was allowed ad libitum. After 8 weeks, five mice in each group were sacrificed, and whole blood was collected from abdominal vena cava of mice into EDTA and heparin-treated evacuated Venoject tubes (Terumo, Leuven, Belgium). Blood was cooled on ice, and 400 μL of blood was centrifuged (1000× *g*, 5 min). The supernatant (plasma) was collected in a new tube and used to measure lipase activity (Figure 2A). The remaining 20 mice in each group (five mice each group) were orally administered with glycidyl oleate dissolved in soybean oil at 0, 0.1, 0.5, and 1.0 mmol/kg b.w. Then, 24 h after oral administration, the mice were dissected, and whole blood was collected in a blood collection tube. Blood was cooled on ice, and 400 μL of blood was centrifuged (1000× *g*, 5 min). The supernatant was removed, and the precipitate was derivatized using FITC (Figure 2B). The study was approved by the Institutional Animal Care and Use Committees of the University (Permit Number: 215309 [date of approval: 30 March 2021]).

### 2.4. Glycidol–Hemoglobin Adduct (diHOPrVal) Measurement

In this study, a modified Edman degradation method was used for the determination of glycidol-Hb adducts. This method uses FITC as the Edman reagent to cleave the N-terminal valine of the chemical-Hb adducts as a fluorescein thiohydantoin (FTH) derivative, and the analyte (DHP-Val-FTH) is measured by LC-MS/MS. The modified Edman decomposition method is called the “adduct FI*R*E procedure™” because it utilizes modified Edman decomposition to measure the adducts (*R*), which is the product of electrophilic addition reactions using FITC reagents [22,23].

#### 2.4.1. Human Blood Sample

Whole blood (250 μL) was alkalized with 1 M KHCO_3_ (20 μL), and the internal standard glycidol-L-Valine-*d*7 adduct fluorescein thiohydantoin (DHP-Val-*d*7-FTH, 10 ppm) [12] was added. FITC (5 mg, 13 μmol) dissolved in *N*,*N*-dimethylformamide (30 μL) was added to the mixture, which was then heated at 37 °C for 18 h in orbital shaker-incubator (ES-20; Biosan, Riga, Latvia) with shaking at 190 rpm.

#### 2.4.2. Animal Blood Sample

Plasma was removed from the whole blood, and 800 μL of saline was added to the precipitate. Then, the blood sample was centrifuged (1000× *g*, 5 min) and washed three times. Afterward, it was stored at −80 °C until use. The lysed blood sample (300 µL) was alkalized with 1 M potassium bicarbonate (20 μL), and the internal standard DHP-Val-*d*7-FTH (10 ppm) was added. FITC (5 mg, 13 μmol) dissolved in *N*,*N*-dimethylformamide (30 μL) was added to the mixture, which was then heated at 37 °C for 18 h in orbital shaker-incubator (ES-20; Biosan) with shaking at 190 rpm.

### 2.5. Solid-Phase Extraction

After FITC derivatization, acetonitrile (1.4 mL) was added to the sample. Next, the protein was precipitated with slow stirring and then centrifuged (10 min at 14,000 × *g*). The supernatant (1.5 mL) was alkalized with 1 M ammonium hydroxide (25 μL). SPE mixed-mode anion exchange cartridges (Oasis MAX) were preconditioned with 1 mL of acetonitrile. The supernatant was loaded onto the column. Then, each 2 mL of acetonitrile was washed with H_2_O and 0.5% aqueous cyanoacetic acid methanol. The analytes were eluted with 0.25% cyanoacetic acid in acetonitrile (1.4 mL). The solvent was evaporated to dryness under a gentle stream of air, and the solid residue was dissolved in 1.0% formic acid/acetonitrile (80 μL, 1:1, *v/v*) prior to analysis. The blood globin level was measured using a HemoCue Hb 201^+^ analyzer (Angelholm, Sweden).

### 2.6. Detection of Hb Adduct by LC–MS/MS

The LC–MS/MS system consisted of an ACQUITY UPLC system interfaced to a Xevo TQ-S instrument (Waters Corporation, Milford MA, USA) with an electrospray ionization source. An L-Column2 ODS was used (2 μm, 2.1 mm × 100 mm; Chemicals Evaluation and Research Institute, Japan, Tokyo, Japan). The mobile phase consisted of (A) 0.1% formic acid in H_2_O/acetonitrile (4:1, *v/v*) and (B) 0.1% formic acid in H_2_O/acetonitrile (1:4, *v/v*). A gradient was applied from 0% B to 20% B in 3 min. The injection volume was 5 μL, and the flow rate was 0.2 mL/min. The mass spectrometer with detection conditions was set as follows: capillary voltage: 3.00 kV; cone voltage: 40 V; desolvation gas flow: 800 L/h; cone gas flow: 150 L/h; nebulizer gas flow: 7.0 L/h; collision energy: 50 eV; desolvation temp: 200 °C. Analysis of processed samples was performed in the positive ion mode using multiple reaction monitoring in accordance with the following transitions: diHOPrVal-FTH, *m/z* 563→390; internal standard DHP-Val-*d*7-FTH, *m/z* 570→390. The limit of detection was defined as the peak height three times the noise. Each target was evaluated by analyzing a calibration sample of five concentrations, including the internal standard (r > 0.999).

### 2.7. Measurement of Lipase Activity

Lipase activity was measured by using the Lipase Kit S (DS Pharma Biochemical Co., Osaka, Japan). Plasma was diluted 50-fold in saline solution, and each sample was divided into two parts: test sample (A) and blank sample (B). After mixing 1 mL of chromogenic solution and 50 µL of diluted plasma, 20 µL of esterase inhibitor solution was added, mixed, and preincubated at 30 °C for 5 min. In the testing sample (A), 100 µL of substrate solution was added, mixed, and incubated at 30 °C for 30 min. After incubation, 2 mL of stop regent was added. For the blank sample (B), 100 µL of substrate solution was added and mixed. The absorbance of the supernatant was measured at 412 nm, and activity was determined as follows:

Lipase activity (IU/mL) = 0.147 × dilution rate × OD_412_.

### 2.8. Statistical Analysis

Results were analyzed using Microsoft Excel 2019 (Microsoft, Redmond, WA, USA) with one-way ANOVA, followed by Dunnett tests. The significance level was set at *p* < 0.05, and all experiments were repeated at least three times.

## 3. Results

### 3.1. Levels of diHOPrVal in the Blood of Humans Fed with Charcoal-Grilled Pork

**Figure 3:**
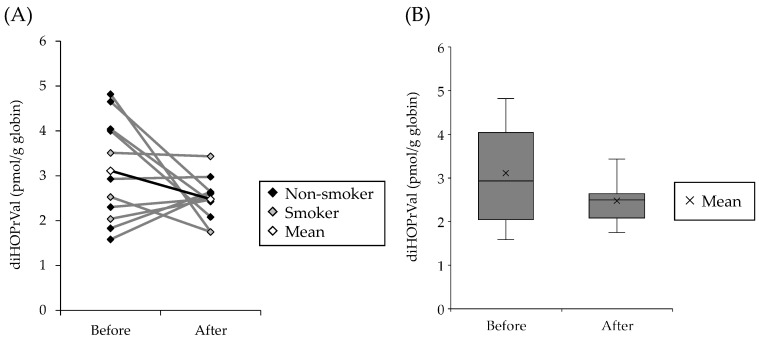
Distribution of individual glycidol-Hb adducts diHOPrVal levels in humans before and after ingestion of pork cooked over charcoal fire. (**A**) Closed rhombus represents individual diHOPrVal. Open rhombus represents average values of diHOPrVal in each group. (**B**) diHOPrVal distribution is illustrated as a box plot showing the median adduct levels with 25, 50, and 75th percentiles presented as vertical boxes and error bars.

The effects of dietary exposure of GEs to diHOPrVal levels in human blood were investigated. As a high-concentration intake source of GEs, pork cooked over charcoal fire (over 400 °C for 3 min) was used as a test meal. The amount of GEs in pork belly heated over charcoal fire at 400 °C for 3 min was 4.8 ± 0.4 mg/kg (glycidyl palmitate, 1.21 ± 0.09 mg/kg; glycidyl stearate, 0.61 ± 0.07 mg/kg; glycidyl oleate, 2.16 ± 0.17 mg/kg; glycidyl linoleate, 0.78 ± 0.07 mg/kg; and glycidyl linolenate, 0.04 ± 0.01 mg/kg). The amount of GEs in cooked pork used as the test meal was 4.8 ± 0.4 mg/kg, and the amount of GEs was approximately 300 µg/day. Figure 3A shows the amount of diHOPrVal in human blood before and after consumption of charcoal-grilled pork. The amount of diHOPrVal before ingesting charcoal-grilled pork was 3.11 ± 1.10 pmol/g globin, which slightly decreased to 2.48 ± 0.47 pmol/g globin after 5 days of ingestion (Figure 3B). The consumption of charcoal-roasted pork for 5 days did not increase diHOPrVal in human blood but rather showed a decreasing trend.

### 3.2. Lipase Activity in Mice Fed High-Fat Diet

**Figure 4:**
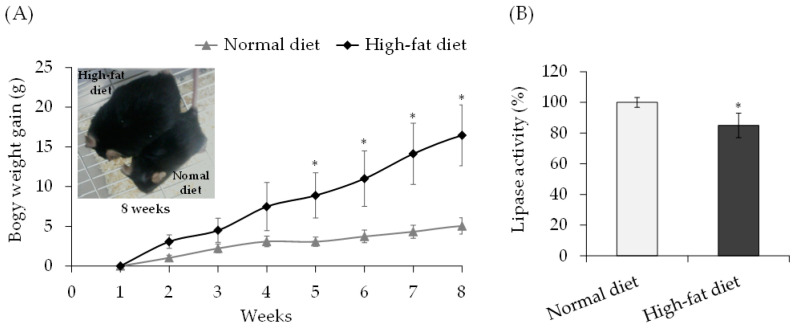
Lipase activity in mice fed a high-fat diet. (**A**) Body weight changes in mice fed normal and high-fat diets. (**B**) Comparison of lipase activity in mice fed normal and high-fat diets. The values represent the mean ± standard deviation (SD) of five independent experiments on five mice. * The significance level was *p* < 0.05 compared with normal diet.

Lipase activity was measured in C57/BL6J mice fed a HFD for 8 weeks. Mice were fed a HFD, and body weight gain was observed, with the HFD group gaining significantly more body weight than the control group (Figure 4A). On day 60 of consuming the high-fat sample, the lipase activity of the blood of the control and HFD groups was measured. When the lipase activity of the control group was 100%, the lipase activity of the HFD group was 85%, showing a significant difference between the two groups (Figure 4B).

### 3.3. Relationship between Decreased Lipase Activity and the Release of Glycidol from GEs in Mice

**Figure 5:**
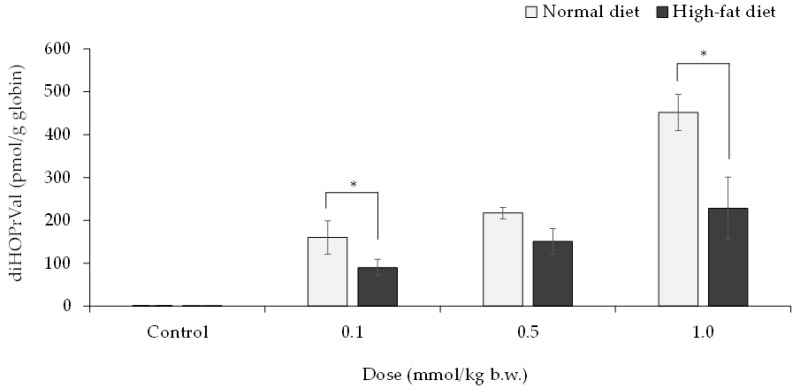
Effect of high-fat diet on glycidol–Hb adduct diHOPrVal formation. GEs (0, 0.1, 0.5, and 1 mmol/kg b.w.) were administered orally on day 59 of the high-fat diet, and diHOPrVal blood was measured by LC–MS/MS. The values represent the mean ± standard deviation (SD) of independent experiments on five mice. * The significance level was *p* < 0.05 compared with normal diet.

GEs (0, 0.1, 0.5, and 1 mmol/kg b.w.) were orally administered to mice on day 59 of the HFD, and blood samples were collected after 24 h to quantify diHOPrVal in the blood. The results showed a concentration-dependent increase in diHOPrVal production in both GEs groups. Compared with the control group, the HFD group had significantly lower amounts of diHOPrVal (Figure 5).

## 4. Discussion

It has been reported that GEs in edible oils are converted to glycidol, a genotoxic and carcinogenic substance, by the action of lipase in vivo [1,2]. The conversion of GEs to glycidol raises concerns about the potential risks of GEs. Genotoxicity of glycidol have been reported in several reports. Glycidol showed mutagenicity with or without a metabolic activation using the Ames test [6]. Micronucleus induction has been confirmed in the bone marrow cells of ICR mice exposed to glycidol using micronucleus test [4]. Oxidative DNA damaging potency of glycidol in the comet assay was reported using Fpg-modified comet assay [5]. Therefore, diHOPrVal, the Hb adduct of glycidol, has been used as a biomarker that can assess exposure to GEs and glycidol [10,11,12,13]. Since DAG oil has higher concentrations of GEs than other commercial cooking oils, it is believed that glycidol exposure may have increased in DAG oil consumers [24,25]. When approximately 36 g of commercial palm fat (8.7 mg/kg of glycidol), which is relatively high in ester-bound glycidol, was fed once daily for 4 weeks to 11 healthy subjects (individual intake ranging from 2.7 to 5.2 μg/kg body weight per day), diHOPrVal increased from 4.0 to 12.2 pmol/g globin [26]. However, Honda et al. reported no significant difference in diHOPrVal levels between a DAG oil user (assuming a daily intake of about 10 (0.8–19.9) g of DAG oil containing a high concentration of 269 µg/g of GEs [27]) and non-user [15,16]. In the present study, ingestion of charcoal-grilled pork containing a large amount of GEs two times a day (approximately 300 μg/day) for 5 days did not significantly increase and tended to slightly decrease diHOPrVal levels in the blood compared with the normal diet (Figure 3). The decrease in diHOPrVal levels could not be estimated, although food records other than the test meal and smoking habits were considered. Possible reasons for the decrease in diHOPrVal levels included the following. In this experiment, subjects consumed cooked pork with high iron content [28]. In addition, pork contains high levels of L-carnitine, which enhances the action of hematopoietic factor erythropoietin [29,30]. Therefore, it was hypothesized that consumption of pork stimulates the hematopoietic system to produce new erythrocytes. It is also possible that continuous ingestion of the chemicals in cooked pork enhances the expression of detoxification enzymes and the ability to excrete glycidol [31]. For these reasons, the amount of glycidol hemoglobin adducts after consumption of cooked pork may have decreased compared to before consumption. Therefore, factors related to the formation and maintenance of Hb adducts are important in investigating the cause of the discrepancies in the exposure assessment of GEs-containing diets.

Although several factors influencing the Hb adduct formation have been investigated [32], the contribution of lipase activity has not been evaluated. The bioavailability of glycidol from GEs have been evaluated in rats, and the amount of diHOPrVal in the blood was equivalent between groups receiving equimolar doses of glycidol or glycidyl palmitate esters [33]. This finding indicated that glycidyl palmitate esters are completely hydrolyzed by lipase to glycidol and palmitic acid. Considering the experimental data obtained in rats for risk assessment, GEs may be completely hydrolyzed in the gastrointestinal tract. On the contrary, when GEs were administered to rats or crab-eating macaques, species differences in the amount of glycidol transferred to plasma were observed [34]. This finding may be related to differences in the activity of lipase, which is involved in the breakdown of lipids in both animal species. Rats have stronger lipase activity from the oral cavity to the stomach than crab-eating monkeys. Therefore, the difference in plasma glycidol levels in both animal groups may be due to the effect of the conversion of GEs to glycidol. The results of this study and these findings indicate that differences in lipase activity and GEs intake may be involved in the formation of diHOPrVal.

Pancreatic lipase is involved in the metabolism of dietary fat, and its secretion is usually increased by lipid intake. Moreover, feeding an HFD decreases mRNA and protein levels of lipoprotein lipase in rats [35]. It was hypothesized that lipase activity decreased because of the increased lipid intake associated with the continuous consumption of charbroiled pork. The consumption of charbroiled pork may have decreased lipase activity and internal exposure to glycidol from its esters. Therefore, in verifying the relationship between lipase activity and the release of glycidol from GEs, the lipase activity and diHOPrVal in blood were measured in C57/BL6J mice fed a HFD for 8 weeks. A previous study showed that when glycidyl oleate was administered to rats at 0.5 and 1.0 mmol/kg b.w., the level of diHOPrVal in the blood at 24 h was 419 ± 50 and 1055 ± 110 pmol/g globin, respectively [36]. Therefore, mice fed with HFD were orally administered glycidyl oleate dissolved in soybean oil at doses of 0, 0.1, 0.5, and 1.0 mmol/kg b.w. Consequently, the lipase activity was significantly decreased in the HFD group compared with the control group (Figure 4B). Furthermore, when GEs were administered to the HFD group, the amount of diHOPrVal decreased compared with the control group (Figure 5). The decreased lipase activity in the HFD group may have weakened the breakdown of GEs to glycidol, thereby decreasing the amount of diHOPrVal. In the human results in this study, the consumption of charbroiled pork may have also reduced lipase activity and internal exposure from glycidol esters. Thus, further study must be conducted to determine if a slight decrease in lipase activity can result in a substantial reduction of the forming diHOPrVal levels.

The action of lipase is essential for the almost complete release of human-ingested GEs during digestion to become free glycidol. However, no unanimous view has been obtained, with reports of increased secretion and activity [35] or suppressed secretion [37] of lipase depending not only on lipid metabolism but also on the effects of exercise, body size, age, and other individual differences. In this study, the findings showed that the activity of lipase in vivo may affect lipid metabolism, and differences in lipase activity may alter the efficiency of glycidol conversion from GEs. Foods may contain ingredients that inhibit lipase activity, and pharmaceuticals such as orlistat and plant-containing ingredients such as plant polyphenols have been reported as lipase inhibitors [38]. The release of glycidol from GEs is dependent on lipase activity; thus, the simultaneous ingestion of lipase inhibitory ingredients and GEs may also affect the formation of diHOPrVal. The efficiency of the conversion of GEs to glycidol when GEs are ingested simultaneously with components that affect lipase activity should also be clarified. In addition, the expression of lipase activity and the metabolism and pharmacokinetics of GEs may differ depending on the intake of GEs from different dietary forms, such as edible oils and various foods. In assessing the risk of GEs to humans, further study must be conducted not only on the exposure amount of GEs from foods and the environment, but also on various influencing factors such as lipase activity, which varies with the amount of lipids in the diet and food components. In future studies on human exposure assessment of GEs, making dietary adjustments before the study, matching fat intake levels during the study, and monitoring lipase activity may be important for the correct interpretation of the study.

## 5. Conclusions

This study examined whether human ingestion of GEs-rich foods increased diHOPrVal, a marker of internal exposure to genotoxic glycidol. Contrary to expectation, the consumption of GEs-rich foods did not increase the levels of diHOPrVal formed from GEs in humans. Considering the influence of lipids in foods, the effect of high fat intake was examined on diHOPrVal levels in mice treated with GEs. The results showed that the levels of diHOPrVal decreased with a reduction in lipase activity. This indicates that the type and amount of ingested food components alter the metabolism of GEs in vivo, thereby changing the toxicity of GEs. This study reveals that various factors, such as daily dietary habits, can alter the potential toxicity of chemicals, providing important and novel findings in the risk assessment of chemical substances.

## Data Availability

The data presented in this work are available in the article.
